# The study protocol: Neuroendocrinology and (epi-) genetics of female reproductive transition phase mood disorder - an observational, longitudinal study from pregnancy to postpartum

**DOI:** 10.1186/s12884-020-03280-5

**Published:** 2020-10-09

**Authors:** Alexandra Johann, Ulrike Ehlert

**Affiliations:** 1 Clinical Psychology and Psychotherapy, University of Zurich, Binzmühlestrasse 14, 8050 Zurich, Switzerland; 2 Swiss National Science Foundation (SNSF), Bern, Switzerland

**Keywords:** Pregnancy, Postpartum, Stress, Depression, Steroid hormones, Psychophysiology

## Abstract

**Background:**

Postpartum depression is considered to be one of the most common health threats during pregnancy and postpartum, affecting not only the woman herself but also the offspring and the whole family system. Evidence for a conclusive etiopathological model with distinct risk and resilience factors is still broadly lacking. Therefore, the aim of the present study is to investigate numerous health-related markers to obtain greater insight into which biopsychosocial profiles render women more vulnerable to PPD or facilitate a healthy transition from pregnancy to postpartum.

**Methods:**

The observational, longitudinal study aims to include a total of 288 physically healthy women, aged 20–45 years. A multitude of relevant parameters, of an (epi-) genetic, endocrinological, physiological and psychological nature, will be assessed over a period of 5 months, following the participants from the 3rd trimester until three months postpartum.

**Discussion:**

The ultimate goal of the present study is to ameliorate mental health care during pregnancy and postpartum, by gaining a better understanding of the underlying biopsychosocial mechanisms that women undergo during the transition from pregnancy to postpartum.

## Background

Depression is projected to become the leading contributor to the global burden of disease worldwide by 2030 [1]. It causes severe impairments to quality of life, and the adverse effects often extend to the whole family system of the affected individual [2]. For instance, maternal depression is associated with unfavorable neonatal birth outcomes, and the offspring can show an up to threefold increased risk of developing a depressive disorder throughout later life [3, 4]. It is well known that women’s lifetime risk of suffering from major depression is twice as high as that of men [5]. This sex difference emerges during early adolescence and leads to a lifetime prevalence of 16.5–21.7% for women in the US and Europe, compared to 8.9–12.7% for men [6, 7].

Even though pregnancy and childbirth mark a period filled with joy and excitement for most women, there is a substantial group of women who are prone to develop mood disturbances throughout pregnancy and the postpartum period. As such, the transition phase from pregnancy to postpartum depicts an exceptionally vulnerable phase in a woman’s reproductive life, given prevalence rates of 10–20% for peri- and postpartum depression [8].

Previous research, including recent work by our own group, points to different trajectories of the onset and development of depressive symptoms during pregnancy and postpartum [9]. In a large, community-based longitudinal online survey (*N* = 687), we found a remarkably high incidence of antenatal and postpartum depressive symptoms (9.2% and 6.3%, respectively) as well as recurrence rates (9.2% and 4.1%, respectively) in healthy women and women with prior depression. It is well established that women with a history of non-puerperal depression show a greater risk of developing depressive symptoms during pregnancy and postpartum [8]. To our knowledge, the aforementioned study was the first in a large Swiss sample to clearly differentiate between recurrent and de novo episodes of depressive symptoms in pregnancy and postpartum. As such, we assume that there might be distinct biopsychosocial profiles, following different etiopathological pathways, which render some women more vulnerable to depression during pregnancy and postpartum compared to women who remain mentally healthy throughout the peripartum period.

Potential mechanisms that render these women more vulnerable might be found in the reported stress-provoked dysregulation of the HPA axis linked to non-puerperal depression [10]. In line with these findings, higher placental corticotropin-releasing hormone (pCRH) levels in mid to late pregnancy have been found to be a strong predictor of postpartum depression (PPD) [11]. A potential explanation may be that the prolonged elevation of pCRH in pregnancy leads to greater residual hypothalamic suppression and HPA axis hypoactivity in women after giving birth [12]. Therefore, women with prior non-puerperal depression might show a phenotype of greater vulnerability to the substantial changes of the HPA axis during pregnancy and postpartum.

A further, hormone-sensitive phenotype has been discussed in previous research in women with a history of premenstrual syndrome/premenstrual dysphoric disorder or prior PPD. These women show a greater risk of developing depressive symptoms during pregnancy and postpartum [13]. Sex steroids rise exponentially during pregnancy, with a sharp drop following delivery and the associated displacement of the placenta [14]. The rapid withdrawal of sex steroids can also be found in other reproductive phases, for example the luteal phase of the menstrual cycle or the perimenopause in later life. It has been shown that some women react particularly sensitively to sudden withdrawal and fluctuations of estrogen and progesterone, resulting in mood disturbances [15, 16]. A notable key player associated with postpartum depression might also be one of the metabolites of progesterone, allopregnanolone: Lower rates in the second and third trimester have been linked to an elevated risk of PPD [17]. Women prone to hormonal sensitivity are also more likely to experience somatization; for instance, hot flushes have been associated with depressive symptoms during pregnancy and in the postpartum period [18].

There is an additional group of women who may show symptoms of depression during pregnancy but find themselves in remission after giving birth. These women might show greater physiological, psychological and psychosocial resources and resilience.

Specific genetic predispositions and epigenetic mechanisms seem to influence the likelihood of suffering from mood disturbances during pregnancy and postpartum, and might even differentiate between women with a history of non-puerperal depression versus women with prior PMS/PPD on a (epi-) genetic level. In non-puerperal and peripartum depression, polymorphisms of the glucocorticoid receptor gene (GR1), the CRH receptor 1 (CRHR1), the short version of the serotonin transporter-linked polymorphic region genotype (5-HTTLPR), as well as the serotonin 2A receptor (HTR2A) and protein kinase C (PRKCB) have been found [19–23]. Distinct biomarkers for PPD focus on polymorphisms that implicate reproductive hormones, e.g. in the estrogen receptor alpha gene, and suggest an enhanced sensitivity to estrogen-based DNA methylation reprogramming [24]. In one study, the development of PPD was predicted with an accuracy of 88% in women with a higher expression change in response to ERα signaling [25]. Research regarding the epigenetic mechanisms of the G protein-coupled estrogen receptor gene 1 is still lacking. So far, despite extensive research, evidence for a conclusive etiological model for PPD, including potentially distinct biopsychosocial profiles and phenotypes, is largely absent, most likely due to the lack of combined assessments of biopsychosocial markers relevant for pregnancy and the postpartum period.

The present study proposes an integrative etiopathological model with distinct trajectories including (epi-) genetic vulnerability, chronic stress or adverse life events, which result in a potential dysregulation of the HPA and HPG axis and their counter-regulation and render some women more sensitive to the fluctuations of sex steroids throughout pregnancy and the postpartum period.

As such, the study seeks to provide an etiopathological model that contributes to explaining the higher prevalence of depressive disorders in women, with the ultimate aim of improving prevention, diagnosis and treatment of peri- and postpartum depression.

##  Methods/design

### Design

The present study will be an observational, longitudinal, single-center, national study. Data will be collected between June 2019 and potentially December 2020, depending on the current situation regarding Covid-19. Pregnant women interested in participating will be screened for eligibility using both 1) an online tool to screen for current depressive symptoms and prior depression with a short questionnaire using validated cut-off scores, and 2) a telephone interview to verify the inclusion criteria. Before completing the screening questionnaire, potential participants will be presented, on an online platform, with the inclusion criteria for study participation, general information about participation and a declaration of consent for the screening. Potential participants will also be provided with a privacy statement, which refers to the relevant privacy aspects of the screening and the online surveys, and to which they must also explicitly agree. The online screening will compromise an examination of the general inclusion and exclusion criteria of the study, the Edinburgh Postnatal Depression Scale (EPDS) and an assessment of prior depressive episodes according to DSM-5 criteria. If the inclusion criteria will be met, participants will be invited to the first of two appointments at the laboratory (lab visit 1) between 34 and 36 weeks of gestation (3rd trimester of pregnancy).

All lab appointments will take place at the lab of the Institute of Psychology at the University of Zurich. During these visits, sociodemographic as well as health- and pregnancy-related information will be obtained. Additionally, the women will respond to psychological self-report questionnaires (see Table 1). As part of the lab visits, participants will undergo the Screening for the Structured Clinical Interview for DSM-IV (SCID-DSM-IV). If necessary, this will be followed by the SCID-IV interview, conducted by a trained clinical psychologist. During both laboratory visits, capillary blood samples (dried blood spots) will be taken from the fingertip using a lancet (Accu-Chek Safe-T-Pro Plus), based on the model of blood glucose measurement in diabetic patients. The blood spots will be absorbed by a special filter paper (Whatman 903) and dried for 3–4 hours at room temperature before being stored. In addition, peripheral physiological measures (blood pressure) will be taken at both laboratory appointments. At the end of the first lab visit, all women will receive a study material package and both oral and written instructions on how to collect the study parameters on their own.

Following lab visit 1, the pregnant women will be asked to provide saliva samples and to complete short questionnaires regarding perceived stress, quality of sleep and mood state on two consecutive weekdays between 34 + 0 and 36 + 0 weeks gestation, and then again as close to the due date as possible (i.e., 40 weeks gestation) in order to capture the steep hormone increase as close to delivery as possible. On the evening before sampling, the women will receive a reminder by email. In total, each participant will collect 32 saliva samples (using Salicaps, IBL) at home, on two consecutive weekdays at 34–36 weeks of gestation, at 40 weeks of gestation, at 4–8 weeks PP, and at 8–12 weeks PP (T1- immediately after awakening; T2–30 min after awakening; T3–45 min after awakening; T4 - in the evening). The women will additionally wear a smart sensor bracelet to collect psychophysiological data during the days of saliva sampling.

**Fig. 1:**
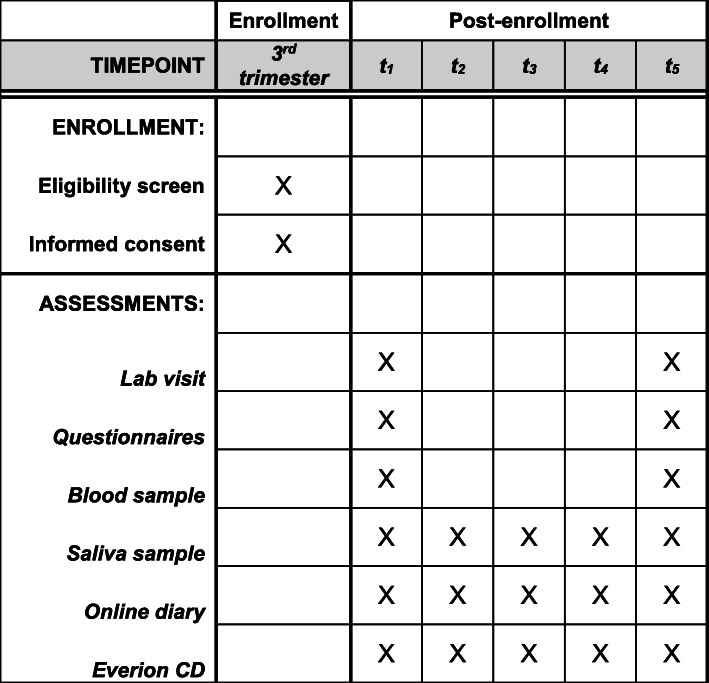
Study design. *t*_*1=34-36 weeks*_*;t*_*2=40 weeks*_*;t*_*3=1-5 days postpartum;*_*t*_*4=4-8 weeks; postpartum;*_*t*_*5=8-12 weeks postpartum*_

At 8–12 weeks postpartum, the women will be invited to a second and final lab visit (lab visit 2). The procedure of the second visit will be comparable to the first (i.e., SCID Screening, dried blood spot sampling, measurement of psychological and psychophysiological parameters). Additionally, birth- and postpartum-specific information will be obtained. All women will be compensated with gift vouchers as well as maternity and infant care products (in total worth approximately CHF 150.-). Moreover, the women will receive a summary of all major research findings. An overview of the study design can be found in Fig. 1.

### Sample

The study will include a total of 288 physically healthy pregnant women aged between 20 and 45 years. Both women who were mentally healthy prior to pregnancy and women with a history of depression according to DSM-5 will be eligible for participation. The women will be examined beginning from the 3rd trimester of pregnancy onwards and followed into the postpartum period 8–12 weeks after giving birth.

The following exclusion criteria will be applied: multifetal gestation; pregnancies achieved through assisted reproductive technology; medical complications (e.g., hypertension, diabetes mellitus, hyperemesis gravidarum, (pre)eclampsia, suspected fetal growth restriction, fetal structural abnormalities); medical condition or surgical intervention that might have affected ovarian function prior to pregnancy or may affect ovarian function during the postpartum period (e.g., polycystic ovary syndrome, endometriosis, breast cancers); current or past history of psychosis, bipolar disorder, posttraumatic stress disorder, eating disorder, substance abuse or dependency; current intake of hormones (e.g., corticosteroids), diuretics, hypertensives, vasodilators; treatment with psychotropic substances within the last 3 months preceding study inclusion; drug use and/or smoking; alcohol intake of more than one standard unit a day; pre-pregnancy body mass index (BMI) > 25 or < 18; protein-restricted diet, and/or regular consumption of soy products.

For the sample size estimation, we performed an a priori sample size estimation analysis using GPower 3.1 [26]. The analysis was computed taking a middle effect size of 0.25 with α = 0.05 and power = 0.95 (1-β a) into account. Since only pregnant women who will give birth to a healthy child will be included into the statistical analyses, and in order to take a drop-out rate of approximately 10% into account, a sample size of *N* = 288 was estimated to obtain comparable subgroups of women with/without prior depression and with/without PPD according to recent prevalence rates.

### Enrollment/informed consent

Recruitment began directly after approval of the proposal by the Ethics Committee of the Canton of Zurich, Switzerland. Data of the 288 study participants will be collected continuously for 30 months. Information about the study will be published on the research team’s website, www.ichbinschwanger.ch, which was developed seven years ago with the goal of providing pregnant women with helpful material and knowledge about the current state of research on psychobiological health during pregnancy. Study information leaflets will be handed out to all pregnant women receiving prenatal medical care at the obstetric departments of all major outpatient clinics of hospitals in and around Zurich, Switzerland. The study information leaflets will also be placed in as many outpatient clinics for gynecology and obstetrics in the Zurich area as possible, and will be handed out to midwives and antenatal classes. Additionally, study information leaflets will be offered to pregnant women visiting psychosocial counseling institutions regarding pregnancy and birth. Moreover, recruitment will also be conducted via various mailing lists, online and newspaper advertisements, specific offers for pregnant women (e.g. prenatal yoga) and social media (e.g. Facebook). Informed consent will be first obtained electronically for the online screening tool, and written informed consent will be obtained prior to any data collection.

### Outcome measures

#### Psychological measures, sociodemographic characteristics and pregnancy-specific parameters

In order to measure relevant psychological parameters, the participants will complete validated German versions of questionnaires regarding the following clusters of variables: psychological state and trait variables such as anxiety, depression and stress; potential risk factors such as early trauma and adverse life events; potential protective factors such as relationship quality; sociodemographic characteristics; and health-, pregnancy-, and birth-specific characteristics. Online data will be collected using the Unipark platform (www.unipark.com/de, certificate ISO 27,001), which is reliably protected from any external access. The BSI-certified data center is subject to requirements of high data protection and safety in conformity with ISO 27,001 based on the IT basic protection. A comprehensive overview of all self-report questionnaires can be found in Table 1.

**Table 1:** Self-report questionnaires

Construct	Assessment instrument	Authors	Items
Postpartum Depression	Edinburgh Postnatal Depression Scale (EPDS)	Cox, J. L., Holden, J. M., & Sagovsky, R., 1987	10
Prenatal stress	Prenatal Distress Questionnaire (PDQ)	Yali & Lobel, 1999; Pluess et al., 2010	12
Premenstrual syndrome	German Version PMS-Questionnaire	Ditzen et al., 2011	30
Reproductive aging	Menopause Rating Scale (MRS II)	Hauser, Schneider, Rosemeier & Potthoff, 1999	11
Anxiety	State-Trait Anxiety Inventory Short Version (STAI-SKD)	Englert, Bertrams & Dickhäuser, 2011	5
Stress, Sleep	Visual analog scales (VAS)	Klinkenberg et al., 2009	5
Anxiety	State-Trait Anxiety (STAI-X)	Laux, Glanzmann, Schaffner & Speilberger	20
Neuroticism	NEO Big Five Short Version	Körner et al., 2008	6
Childhood Traumatic Experiences	German Version Childhood Trauma Screener (CTS)	Grabe et al., 2012	5
Life events	German Version Life Experience Survey (LES)	Sarason et al., 1978; Pluess et al., 2010	55
Self-esteem	Rosenberg Self-esteem Scale (SES)	Collani & Herzberg	10
Daily hassles	ZIPS Subscale (Daily Uplifts)	Bratsikas, Mohiyeddini, Viecelli & Ehlert, submitted	10
Maternal-fetal attachment	Maternal fetal attachment scale (MAFS)	Cranley, 1981; Sjörgen et al., 2004	17
Relationship quality	Couples Satisfaction Index	Funk & Rogge, 2007	4
Social support	Berlin Social Support Scales (BSSS)	Schulz & Schwarzer, 2000	17
Traumatic experiences Lifetime	German Version Trauma History Questionnaire	Green, 1996; Maercker & Bromberger, 2005	24
Birth Anxiety	Birth Anxiety Scale (GAS) Short Version	Lukesch, 1983	25
Perfectionism	Multidimensional Perfectionism Scale (MPS)	Altstötter, Gleich & Bergemann, 2006	35
Impact of Events	Impact of Event Scale (IES) Horowitz	Stadlmayr, Cignacco, Surbek, Büchi	15
Mother-baby interaction	The mother and baby interaction scale	Hovik et al., 2013	9
Chronic stress	Trier Inventory for Chronic Stress, Short Version (TICS-K)	Schulz, Scholz & Becker, 2004	39
Emotions towards motherhood	LIWC2015_Language Manual	Pennebaker et al., 2015	1
Maternal-fetal Attachment	Pictorial Representation of Attachment (PRAM)	Van Bakel et al., 2013	3
Emotional support	Maternal adjustment five months after birth	Lemola et al., 2007	7

**Table 2:** Biological parameters

Source	Label	Parameter
Saliva (SaliCaps)	Endocrine parameter	- Cortisol - Progesterone - Estradiol - Allopregnanolone
Blood (dried blood spots)	Endocrine and inflammatory parameter	- Serotonin - GABA - LH - FSH - Corticotropin-releasing hormone (CRH)
Blood (dried blood spots)	(Epi-) genetic parameter	- ER Alpha - ER Beta - GPER - NR3C1 - CRHR1 - FKBP5
Blood pressure	Periphysiological parameter	- blood pressure
Everion CD - bracelet	Psychophysiological parameter	- heart rate - heart rate variability - skin blood perfusion - resting respiratory rate - activity - steps - skin temperature - barometric pressure - electrodermal activity - blood oxygen saturation

#### Neuroendocrine parameters

Neuroendocrine parameters will be measured using saliva and dried blood spot sampling, with blood obtained from finger prick. With regard to saliva, the HPG hormones of interest will include salivary E, P, and ALLO. Salivary cortisol, the end product of the HPA axis, will also be analyzed.

Saliva samples will be collected via Salicaps (a passive drooling device; IBL International GMBH, Hamburg, Germany). Salivary E, P, and cortisol concentrations will be quantified by luminescence immunoassay with commercial immunoassay kits (IBL-Hamburg, Germany). Analyses will be conducted in our own laboratory. ALLO will be analyzed from the first measurement time point at morning awakening using an enzyme-linked immunosorbent assay (ELISA).

Blood samples will be collected through finger prick. The participants’ finger will be pricked with a sterile, disposable lancet (commonly used by diabetics, Accu-Chek Safe-T-Pro Plus), and up to five drops of blood (about 50 µL per drop) will be spotted onto standardized filter paper (no. 903 Whatman, DBS Protein Saver Card). Serotonin and CRH will be assessed from dried blood samples obtained during lab visit 1 and kept at − 20 °C until analysis [27, 12]. GABA, LH, and FSH will be measured in dried blood spots collected during lab visits 1 and 2 [28]. The filter paper samples will be air-dried after collection and stored in an airtight plastic bag at − 20 °C until analysis. Biochemical analyses will be conducted at our own laboratory and the Cytolab laboratory in Regensdorf, Switzerland. An overview of the assessed biological parameters can be found in Table 2.

#### Genetic and epigenetic parameters

Methylation will be assessed using an established Next Generation Sequencing (NGS) method: medium throughput bisulfite sequencing [29], which has been previously used by our research group in close cooperation with the lab of Prof. Dr. Turecki, McGill University, Canada. The assay (e.g., primer design) for the methylation analyses of the NR3C1, FKBP5, ERα, ERβ, and GPER are already established in the lab, while the assay development for the CRHR1 gene is currently ongoing and will be fully operational by the projected time of analysis.

TagSNPs selected in the candidate genes, according to the previously reported TagSNPs selection procedure [30], and SNPs showing evidence of association, will be included in a custom genotyping array and analyzed at the Genetic Diversity Center (GDC), ETH, Zurich. Variable length tandem repeats (VNTRs) will be analyzed by polymerase chain reaction (PCR) followed by capillary electrophoresis [31].

#### Psychophysiological parameters

To assess relevant psychophysiological data, participants will wear a smart sensor bracelet, the Everion CD, developed by the medical technology company Biovotion (https://www.biovotion.com). The Everion CD is a non-invasive physiological monitor that is CE-certified as a class IIa medical device. It provides 22 parameters and features measured in very short intervals (every second) with medical-grade quality. Measured psychophysiological parameters that are influenced by both the HPG and HPA axis hormones include the following: heart rate (HR), heart rate variability (HRV), resting respiratory rate (RR), skin temperature, electrodermal activity (EDA), skin blood perfusion, steps, activity, barometric pressure, blood pulse wave, and blood oxygen saturation.

The large collected dataset will permit us to address multiple research questions, such as associations between perceived sleep quality, objective amount of sleep, and the development of PPD. Additionally, the sensor bracelet will be able to track participants’ sleep duration and awakening time, which is essential for the statistical analyses and interpretation of the morning awakening response [32]. These data will be transferred to a smartphone app, which the women will download and install on their smartphone upon study enrollment. Besides big data analyses in cooperation with the mobile devices center of the URPP Healthy Aging, the collected data will be investigated with specific algorithms provided by Biovotion. For data security, each woman will receive a unique identification code. Biovotion handles customer data in compliance with the GDPR. Finally, participants’ blood pressure will be assessed during the two lab visits in order to control for cardiovascular parameters in addition to the measurements with the Everion CD bracelet.

### Statistical analysis

The statistical analyses will be conducted using the Statistical Package for the Social Sciences (SPSS 22.0, Armonk, NY, USA) and the open-source software R (the R Foundation for Statistical Computing, Vienna, Austria) amongst others. Multivariate analyses of variance, multivariate regression analyses, cluster analyses, multilevel/mixed models, and mediator and moderator analyses will be used to analyze the neuroendocrine, psychological, (epi-) genetic and physiological parameters [33, 34]. Hypothesis-free data-mining approaches will be used to analyze the large amounts of data collected via the smart sensor bracelet in combination with the repeatedly assessed neuroendocrine and psychological parameters [35]. With these analyses, the associations between biorhythms and depressive symptoms will be explored. To minimize the risk of over-interpreting incidental findings, split-half procedures will be used to compare hypothesis-driven analyses with data-mining models [36]. Moreover, all statistical analyses will be corrected for multiple testing as appropriate. The two-sided significance level will be set at *p* < .05. The study population will be homogenous in terms of pregnancy/PP status (from 34 to 36 weeks of pregnancy until 8–12 weeks PP).

### Study status

**Fig. 2:**
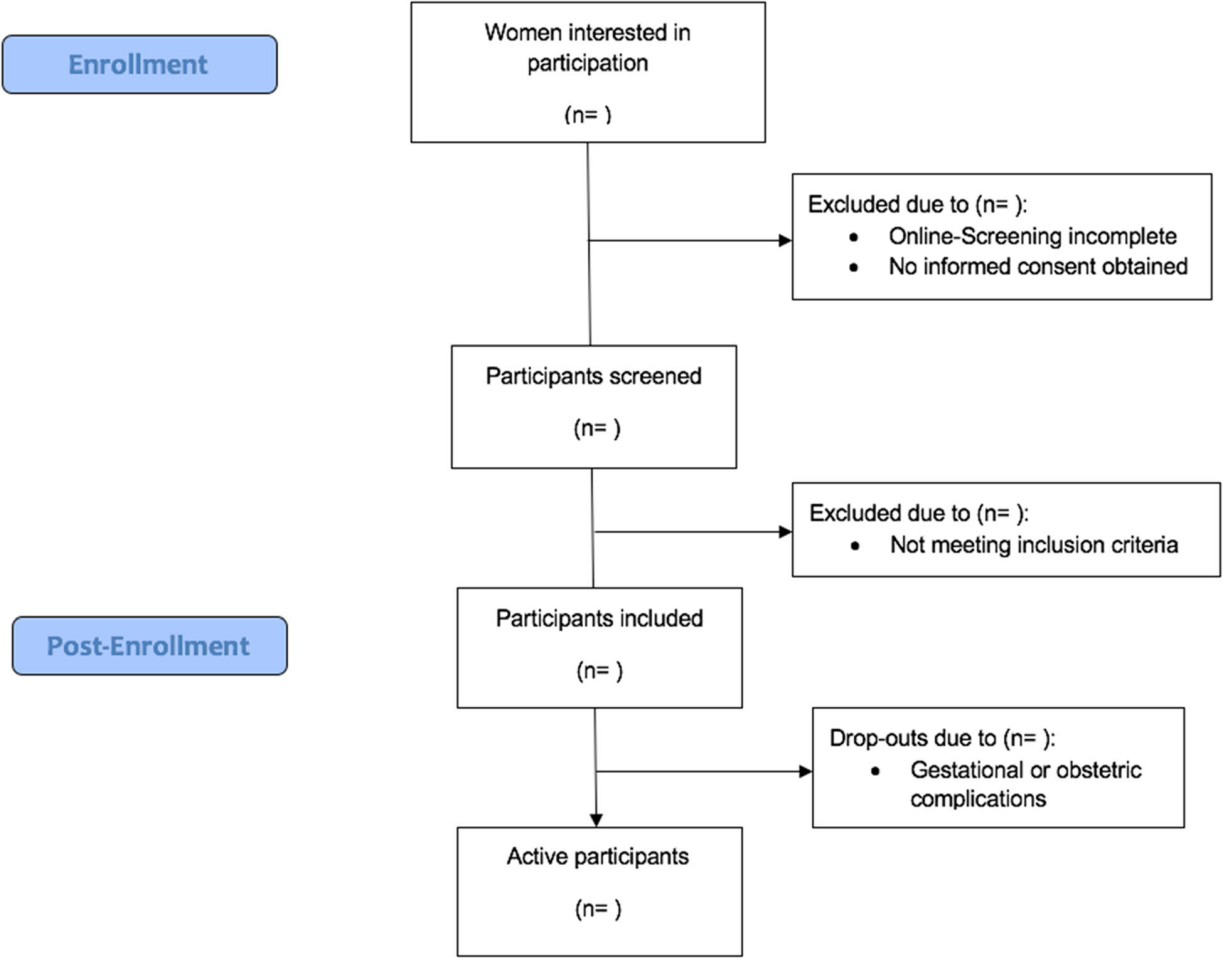
Sample inclusion

From June 2019 to March 2020, a total of 659 women expressed an interest in participating by clicking on the online screening questionnaire, and 150 (22.8%) women completed the online screening questionnaire. After reassessment of the inclusion criteria by telephone, 101 (15.3%) participants were found to be eligible for the study, until a recruitment halt due to Covid-19. Until March 2020, 18 (17.8%) women were reported as dropouts, mostly because of pregnancy- or postpartum-related medical complications. An overview of the inclusion process can be found in Fig. 2.

##  Discussion

The present study aims to provide further insight into the etiopathological mechanisms that render some women more vulnerable to mood disturbances and anxiety throughout pregnancy and the postpartum period. To the best of our knowledge, previous research has mostly focused on either biological or psychological factors, whereas a comprehensive investigation of all relevant factors is still lacking. The main goal is to assess and evaluate specific health-related markers, such as (epi-) genetic factors, hormonal patterns, and psychosocial and physiological parameters that contribute to a healthy transition or maladaptation from pregnancy to postpartum.

Given the prevalence rates and multiple possible negative outcomes for the affected individual and the whole family system, it is of utmost importance to gain deeper insight into the underlying mechanisms of PPD. Previous research has pointed in the direction of possibly different trajectories, meaning that not all women are affected equally by the same hormonal changes or risk factors. Therefore, this study aims to find not only an overall etiopathological model, but also different trajectories of PPD, consisting of specific subgroups of vulnerable women who show a distinct biopsychosocial profile. The strengths of the present study design lie in its longitudinal nature, the application of strict inclusion criteria and the large set of relevant markers being assessed. A further strength is the inclusion of women with prior depression, women with current depression and/or anxiety, and mentally healthy women, which will enable us to follow up different pathways and their underlying mechanisms during pregnancy and the postpartum period. Since distinct (epi-) genetic markers of PPD are still largely missing, we hope to obtain greater insight into possibly relevant gene-environment interactions regarding PPD. To gain objective physiological data, we will use smart sensor bracelets in order to monitor physiological symptoms in addition to self-assessment questionnaires (e.g. regarding sleep and stress). In conclusion, the present study aims to fill the gap in terms of achieving a conclusive etiopathological model regarding PPD by assessing numerous relevant biopsychosocial parameters in a longitudinal design and large sample. This should ultimately enable us to distinguish which women tend to have a high risk for mood disturbances during pregnancy and the postpartum period and thereby enhance prevention, diagnosis and treatment of PPD.

###  Limitations

So far, no declarations can be made, since the study is still ongoing.

## Data Availability

Not applicable.

## Abbreviations

ALLO: Allopregnanolone
BMI: Body Mass Index
BSI: Federal Office for Information Security (Bundesamt für Sicherheit in der Informationstechnik)
CAR: Cortisol Awakening Response
CRH: Corticotropin-Releasing Hormone
DBS: Dried Blood Spots
DNA: Deoxyribonucleic acid
DSM: Diagnostic and Statistical Manual of Mental Disorders
E: Estradiol
ELISA: Enzyme-Linked Immunosorbent Assay
FSH: Follicle-stimulating hormone
GABA: Gamma aminobutyric acid
GDPR: General data protection regulation
HPA: Hypothalamic-pituitary-adrenal axis
HPG: Hypothalamic-pituitary-gonadal axis
IBL: Immuno-Biological Laboratories
ISO: International Organization for Standardization
LH: Luteinizing Hormone (LH)
NGS: Next-Generation Sequencing
P: Progesterone
pCRH: Placental Corticotropin-releasing hormone
PMS: Premenstrual syndrome
PPD: Postpartum depression
PP: Postpartum
SNP: Single nucleotide polymorphism
